# Prevalence of Enteric Fever in Febrile Patients: Clinico-Bacteriological and Antimicrobial Profile in South Delhi, India

**DOI:** 10.7759/cureus.104585

**Published:** 2026-03-02

**Authors:** Sehar Shafiq, Neetu Shree, Jayanthi Gunasekaran, Vineet Jain, Afreen Khan

**Affiliations:** 1 Microbiology, Hamdard Institute of Medical Sciences and Research, New Delhi, IND; 2 Internal Medicine, Hamdard Institute of Medical Sciences and Research, New Delhi, IND

**Keywords:** antimicrobial susceptibility, azithromycin, blood culture, ceftriaxone, enteric fever, fever, fluoroquinolones, salmonella paratyphi, salmonella typhi

## Abstract

Objectives

The main objective of this study is to determine the prevalence and clinical presentation of *Salmonella Typhi* and *Paratyphi* bacteremia in cases of suspected enteric fever, and their antimicrobial-resistance pattern.

Material and methods

Blood specimens were collected for culture from suspected enteric fever patients, along with relevant clinical history and examination results. Culture was performed using the Automated Bac T/Alert 3D (bioMérieux, Marcy-l'Étoile, France) blood culture system. When flagged positive, isolation was done on Blood agar and MacConkey agar, followed by identification using standard bacteriological operating procedures, along with Vitek 2 (bioMérieux, Marcy-l'Étoile, France) and serotyping. Antimicrobial susceptibility testing was done by Vitek 2, and additionally by the disc diffusion method.

Results

Of 16,052 blood samples, 5.66% (n = 909) were positive, of which 46.64% (n = 424) were typhoidal *Salmonella* (*S. Typhi* and *S. Paratyphi*). Of these, 83.25% (n = 353) isolates were *S. Typhi* and 16.74% (n = 71) were *S. Paratyphi* *A* and *B*. Predominant clinical features included fever 100% (n = 424), vomiting/nausea 66.98% (n = 284), abdominal pain 68.86% (n = 291), and diarrhea 67.92% (n = 288). Typical features, i.e., rash, were noticed in only 2.59% (n = 11) of cases, whereas coated tongue was observed in 37.73% (n = 160) of patients. Among isolates of *S. Typhi* (n = 353), multi-drug resistance (MDR) was noted in 3.68% (n = 13) of isolates; resistance rates of 9.06% (n = 32), 4.53% (n = 16), and 3.68% (n = 13) were noted for ampicillin, chloramphenicol, and co-trimoxazole, respectively. No resistance was observed for ceftriaxone or carbapenems. Azithromycin resistance was noted in 2.83% (n = 10) of *S. Typhi* isolates. None of the *S. Paratyphi* isolates were MDR. High resistance to ciprofloxacin was observed among *Salmonella* isolates, with 72.23% (n = 255) and 88.73% (n = 63) noted for *S. Typhi* and *S. Paratyphi*, respectively.

Conclusion

Among the total bacteremia cases, almost half were due to typhoidal *Salmonella*, which highlights the importance of blood culture for all suspected enteric fever cases. As fever and abdominal pain were the predominant symptoms among the confirmed cases of enteric fever in this study, these symptoms overlap with those of other conditions causing fever. This makes specific testing for enteric fever very important for accurate diagnosis, which can help avoid complications and support complete treatment, thereby preventing carrier states and antimicrobial resistance (AMR). AMR among typhoidal isolates is becoming a major threat to management, with increasing resistance to commonly used antimicrobials. Routine surveillance is essential to understand the locally prevalent antibiogram patterns.

## Introduction

Typhoid fever, often termed enteric fever, is a multisystemic illness of serious public health concern, especially in impoverished and emerging nations, including India, with a high disease burden (more than 50% of the world), resulting in significant morbidity and mortality [1]. In 2021, enteric fever affected 9.3 million people worldwide, resulting in 107.5 thousand fatalities [2]. The age-standardized incidence rate fell from 152/100,000 person-years in 2017 to 128/100,000 person-years in 2021, while the death rate dropped from 1.87/100,000 person-years to 1.50/100,000 person-years [2]. There were significant geographical disparities, with South Asia accounting for the most cases and deaths [2]. In 2021, the age-standardized incidence of enteric fever exceeded the “high burden” threshold of 100/100,000 person-years in 23 countries [2].

Enteric fever is still endemic in India due to various factors, including lack of hygiene, sanitation facilities, education and awareness, overcrowding, etc., related to socioeconomic conditions. The consistency of susceptibility patterns is no longer valid, leading to rising antimicrobial resistance (AMR) and posing a major public health challenge to physicians and clinical microbiologists. *Salmonella enterica* serotypes Typhi and Paratyphi are responsible for enteric fever (typhoid and paratyphoid), a multisystemic illness transmitted via the fecal-oral route through contaminated food, water, or fomites [3]. The clinical presentation of enteric fever includes step-ladder fever, headache, abdominal pain, anorexia, malaise, rose spots, and hepatosplenomegaly in severe cases. However, due to widespread antibiotic use, classical features are now infrequently observed [4]. Chronic carriage, with risk factors such as biliary disease, achlorhydria (reduced gastric acid), female sex, age >50 years, and inadequate or delayed treatment, can perpetuate transmission. Risk factors for severe disease include immunosuppression, comorbidities such as diabetes or HIV, and gastric acid suppression [5].

Antibiotics are the mainstay of treatment, and commonly used antimicrobials include third-generation cephalosporins and azithromycin. Fluoroquinolones, commonly used in the past, are now increasingly associated with resistance [6]. The problem becomes graver and more complicated with increasing AMR among typhoidal *Salmonella* isolates, reducing the available treatment options and making patient management difficult. Despite the availability of effective antibiotics, the prevalence of multidrug-resistant (MDR) and extensively drug-resistant (XDR) *S. Typhi* strains is rising, presenting a major challenge to treating physicians worldwide [7,8]. In India, MDR (co-resistance to chloramphenicol, ampicillin, and co-trimoxazole) among *S. Typhi* isolates has declined and is now relatively uncommon, reported at approximately 2% (0%-3%) in recent genomic surveillance studies [9]. In contrast, true XDR *S. Typhi*, defined as MDR plus resistance to fluoroquinolones and third-generation cephalosporins, has been documented only sporadically in India and remains rarely reported, typically in isolated cases rather than as a widespread phenomenon [9]. Empirical selection of appropriate antimicrobial therapy, coupled with constant efforts to evaluate changing patterns of antibiotic susceptibility, forms the foundation of a multipronged strategy for the management of enteric fever. Understanding the local epidemiology and resistance patterns of typhoidal *Salmonella* is essential for guiding empirical therapy and public health interventions.

Therefore, this study was undertaken to assess the clinico-bacteriological profile of typhoidal *Salmonella* in bacteremia cases among febrile patients at a tertiary care hospital in South Delhi, India, with a focus on the antimicrobial susceptibility profile. The findings aim to inform and update clinicians and policymakers for the effective management and control of enteric fever in endemic settings.

## Materials and methods

Aims and objectives

This study aimed at determining the prevalence of *S. Typhi* and *S. Paratyphi* bacteremia in cases of suspected enteric fever. The study objectives were to analyze the AMR pattern among isolates of *S. Typhi *and *S. Paratyphi* in cases of bacteremia, and to determine the profile of clinical presentation in cases of typhoidal/paratyphoidal bacteremia.

Study type, duration of study, and venue

A cross-sectional study was conducted over 24 months (January 2023-December 2024) in the Department of Microbiology at Hamdard Institute of Medical Sciences and Research, New Delhi, India.

Ethical Clearance

Ethical approval was obtained from the Institutional Ethics Committee (Hamdard Institute of Medical Sciences and Research), vide approval no. HIMSR/IEC/0058/2022.

Study population

Sample Size

Sample size calculation: As per prevalence data from various authors, ranging generally between 10% and 40%, the average prevalence was considered as 25%, which is also applicable to our area. The sample size was calculated to be 228 using the following formula: $n = \frac{Z_{1-\alpha/2}^2 \cdot p (1-p)}{d^2}$. Proportion of subjects with the target condition (p) = 0.25 (25%); d = precision (tolerated margin of error), i.e., 0.05 (5%); type I error (α) = 5%; Z_(1−α⁄2)_ = 1.96.

Based on the formula given above and using the mentioned values, the sample size required is: $(1.96)^2 \times 0.25 \times (1-0.25) / (0.05)^2 = 228$. Thus, with a 95% confidence interval, the proposed sample size for the study is 228. However, we had 424 confirmed isolates of typhoidal *Salmonella* included in this study.

Inclusion criteria: All suspected patients of enteric fever whose samples were sent for culture and antimicrobial susceptibility testing.

Exclusion criteria: Duplicate samples and cultures positive for non-typhoidal *Salmonella*.

Sample processing

Blood specimens were aseptically collected from febrile patients attending the in-patient department (IPD) and out-patient department (OPD). Samples were processed for blood culture using the Automated Bac T/Alert 3D system (bioMérieux, Marcy-l'Étoile, France). Positive cultures were subcultured, and isolates were identified using standard bacteriological procedures, including colony morphology on MacConkey agar, Gram staining, and biochemical reactions. Isolates of *S. enterica* serotypes Typhi and Paratyphi from positive cultures were further confirmed and serotyped using specific antisera. Antimicrobial susceptibility testing was performed using the Vitek-2 automated system (bioMérieux, Marcy-l'Étoile, France) and additionally by the Kirby-Bauer disk diffusion method on Mueller-Hinton agar for azithromycin susceptibility, following the latest Clinical Laboratory Standards Institute (CLSI, 2023) guidelines for studying antimicrobial susceptibility profiles.

Data collection

Patient demographic details were recorded using the patient requisition form after obtaining consent. Details such as name, age, sex, residential address, and date of registration, as well as laboratory details, including the date of sample collection, date of sample processing, date of reporting, and test results, were recorded. Positive blood culture isolate identification and antimicrobial susceptibility testing results were also recorded.

Data analysis

All collected details were documented in Excel (Microsoft® Corp., Redmond, WA, USA), and the results were analyzed using IBM SPSS Statistics for Windows, Version 26 (Released 2018; IBM Corp., Armonk, NY, USA), based on the study objectives. Data were summarized using frequencies and percentages. A Chi-square test was used to determine the significance of the distribution of *Salmonella* isolates. A p-value <0.05 was considered statistically significant. Adjusted odds ratios and 95% confidence intervals were also applied where required.

## Results

Of 16,052 blood samples collected from patients over a period of 24 months, 909 samples yielded significant pathogens, with an overall blood culture positivity rate of 5.66% (909/16,052; 95% CI: 5.31-6.01%). Typhoidal *Salmonella* yielded an overall prevalence of 2.64% (424/16,052; 95% CI: 2.40-2.90%). Typhoidal *Salmonella* accounted for 46.64% (n = 424) of bacteremia cases, followed by *Escherichia coli*, 17.60% (n = 160), and *Staphylococcus* spp., 13.97% (n = 127). Other relevant pathogens included *Acinetobacter* spp., 6.27% (n = 57); *Klebsiella* spp., 4.51% (n = 41); *Candida* spp., 3.80% (n = 28); *Enterococcus* spp., 3.19% (n = 29); and *Pseudomonas* spp., 2.20% (n = 20), in descending order of frequency (Table 1).

**Table 1:** Distribution of blood culture isolates among enteric fever suspects Values are expressed as numbers and percentages of total positive cultures.

Positive patients	Frequency (n = 909)	Percentage (%)
Salmonella Typhi	353	38.83%
Salmonella Paratyphi	71	7.81%
Escherichia coli	160	17.60%
*Staphylococcus *species	127	13.97%
*Acinetobacter *species	57	6.27%
*Klebsiella *species	41	4.51%
*Enterococcus *species	29	3.19%
*Pseudomonas *species	20	2.20%
*Citrobacter *species	6	0.66%
*Streptococcus *species	7	0.77%
Others	10	1.10%
*Candida *species	28	3.08%

Among *Salmonella* isolates, *S. Typhi* was predominant, 83.25% (n = 353), followed by *S. Paratyphi* A, 15.33% (n = 65), and *S. Paratyphi* B, 1.41% (n = 6). A critical observation is that there is a high number of isolates in the IPD, particularly for *S. Typhi* (85.27%), indicating that a vast majority of these enteric fever cases presented with clinical severity requiring hospitalization. The p-value of 0.000 signifies a statistically significant difference in the distribution of these isolates between outpatient and inpatient settings, highlighting the high disease burden (Tables 2-3).

**Table 2:** Distribution of typhoidal Salmonella isolates in OPD and IPD patients A significant association was observed between isolate type and patient category (Chi-square test, p < 0.001). Values are expressed as numbers (percentages). “*” symbol denotes there is a significant association. OPD, Out-Patient Department; IPD, In-Patient Department

Isolates	OPD, N1 (%)	IPD, N2 (%)	Total (n = 424)	Percentage (%)	p-value
Salmonella Typhi	52 (14.73)	301 (85.27)	353	83.25%	0.000^*^
Salmonella Paratyphi	25 (35.21)	46 (64.79)	71	16.74%	
Salmonella Paratyphi A			65	15.33%	
Salmonella Paratyphi B			6	1.41%	

**Table 3:** Association between type of Salmonella infection and patient visit category (IPD vs. OPD) with risk estimate (N = 424) Crosstabulation showing the distribution of *S. Typhi* (n = 353) and *S. Paratyphi* (n = 71) among inpatients (IPD) and outpatients (OPD). Odds ratio analysis demonstrated that *S. Typhi* infection was significantly more associated with IPD visits compared to *S. Paratyphi* (OR = 3.146; 95% CI: 1.781-5.557; p < 0.001). Values are expressed as counts.

Risk Estimate			
	Value	95% Confidence Interval	
		Lower	Upper
Odds ratio for infection *(Salmonella Typhi/Salmonella Paratyphi)*	3.146	1.781	5.557
For cohort visit = IPD	1.316	1.103	1.571
For cohort visit = OPD	0.418	0.280	0.626
N of valid cases	424	-	-

Age-wise distribution of typhoidal *Salmonella* isolates

Culture-confirmed enteric fever cases were most frequent in the 10-20-year age group, 33.09% (n = 140), followed by <10 years, 31.37% (n = 133), and 20-30 years, 26.42% (n = 112). Fewer cases were reported in the 30-40 years (6.83%, n = 29), 40-50 years (1.65%, n = 7), and >50 years (0.70%, n = 3) groups. No significant association was found between *Salmonella* species and age group (p = 0.264) (Table 4).

**Table 4:** Distribution of typhoidal Salmonella isolates among different age group Distribution of *S. Typhi* (n = 353) and *S. Paratyphi* (n = 71) across age groups. Most cases occurred in 10-20 years (33.09%), followed by <10 years (31.37%). No significant association was found between age and serovar (χ² test, p = 0.264). Values are expressed as numbers (percentages).

Age	*Salmonella Typhi *(n = 353)	*Salmonella Paratyphi* (n = 71)	Total Positive (n = 424, 46.64%)	p-value
<10 years	114	19	133 (31.37%)	0.264
10-20 years	119	21	140 (33.09%)	
20-30 years	91	21	112 (26.42%)	
30-40 years	23	6	29 (6.83%)	
40-50 years	4	3	7 (1.65%)	
≥50 years	2	1	3 (0.70%)	
Total	353	71	424	

Gender-wise distribution of *S. Typhi* and *S. Paratyphi* isolates

The overall male-to-female ratio among enteric fever patients was 1.51:1. However, no statistically significant association was observed between gender and the type of *Salmonella* isolated (p = 0.253) (Tables 5-6).

**Table 5:** Distribution of S. Typhi and S. Paratyphi isolates among different genders Distribution of *S. Typhi* (n = 353) and *S. Paratyphi* (n = 71) among male and female patients. No significant association was observed between gender and serovar distribution (Chi-square test, p = 0.253). Values are expressed as numbers (percentages).

Gender	*Salmonella Typhi *(n = 353)	*Salmonella*​​​​​​​* Paratyphi* (n = 71)	Total	p-value
Male	208 (58.92%)	47 (66.19%)	255	0.253
Female	145 (41.07%)	24 (33.80%)	169	
Total	353	71	424	

**Table 6:** Association between Salmonella infection type and gender with risk estimate (N = 424) Crosstabulation of *S. Typhi* (n = 353) and *S. Paratyphi* (n = 71) according to gender. Odds ratio analysis showed no significant association between gender and type of infection (OR = 0.733; 95% CI: 0.429-1.251; p > 0.05). Values are expressed as counts.

Risk Estimate			
	Value	95% Confidence Interval	
		Lower	Upper
Odds ratio for infection (*Salmonella* *Typhi/Salmonella Paratyphi*)	0.733	0.429	1.251
For cohort gender = male	0.890	0.738	1.074
For cohort gender = female	1.215	0.857	1.722
N of valid cases	424	-	-

Clinical features

Most patients (53.30%, n = 226) had fever lasting ≥7 days, while 46.69% (n = 198) had fever for a duration <7 days. Common accompanying symptoms are described in the following table. Complications were rare (1.41%, n = 6), including five cases of intestinal obstruction and one case of acute pyelonephritis with early shock (Table 7). Most patients were from lower socio-economic backgrounds near South Delhi slums, with >90% unimmunized or unable to recall prior typhoid vaccination. Laboratory data from typhoid and paratyphoid patients showed low hemoglobin (<9 mg/dL) in 32 (7.54%), leukopenia (<4000 cells/μL) in 22 (5.18%), and leukocytosis (>11,000 cells/μL) in 33 (7.78%). No mortality was observed among our patients.

**Table 7:** Clinical features among patients with enteric fever Drug resistance profile of *Salmonella Paratyphi* shows high resistance to ciprofloxacin (88.73%) and nalidixic acid (100%), with preserved sensitivity to chloramphenicol and cotrimoxazole (100%), and high sensitivity to meropenem (97.18%). Values are expressed as numbers (percentages) of isolates categorized as resistant, intermediate, or sensitive.

Clinical Features	Frequency	Percentage
Fever	424	100%
Vomiting/nausea	284	66.98%
Abdominal pain	291	68.86%
Diarrhea	288	67.92%
Constipation	48	11.32%
Skin rash	11	2.59%
Headache	39	9.2%
Decreased appetite	173	40.80%
Coated tongue	160	37.73%
Pallor	152	35.84%
Complications	6	1.41%

AMR pattern

Among *S. Typhi* isolates (n = 353), resistance was observed for ampicillin, 9.06% (n = 32); chloramphenicol, 4.53% (n = 16); and co-trimoxazole, 3.68% (n = 13) (Table 8). In contrast, *S. Paratyphi* isolates (n = 71) showed higher ampicillin resistance (22.53%) but no resistance to chloramphenicol or co-trimoxazole (Table 9). No resistance was detected to third-generation cephalosporins (ceftriaxone) or meropenem among either species.

**Table 8:** Antimicrobial susceptibility pattern of Salmonella Typhi (n = 353) Susceptibility profile of *S. Typhi *isolates shows high resistance to ciprofloxacin (72.23%) and nalidixic acid (94.61%), with high sensitivity to third-generation cephalosporins and meropenem (100%). Values are expressed as numbers (percentages) of isolates classified as resistant (R), intermediate (I), or sensitive (S).

Drug	Resistant (R)	Intermediate (I)	Sensitive (S)
Ampicillin	32 (9.06%)	0	321 (90.93%)
Azithromycin	10 (2.83%)	0	343 (97.17%)
Chloramphenicol	16 (4.53%)	0	337 (95.47%)
Cotrimoxazole	13 (3.68%)	0	340 (96.31%)
Ceftriaxone	0 (0%)	7 (1.98%)	346 (98.01%)
Cefotaxime	0 (0%)	1 (0.28%)	352 (99.71%)
Ciprofloxacin	255 (72.23%)	89 (25.21%)	9 (2.54%)
Meropenem	0	0	353 (100%)
Nalidixic acid	334 (94.61%)	0	19 (5.38%)

**Table 9:** Antimicrobial susceptibility pattern of Salmonella Paratyphi (n = 71) Drug resistance profile of *S. Paratyphi* shows high resistance to ciprofloxacin (88.73%) and nalidixic acid (100%), with preserved sensitivity to chloramphenicol and cotrimoxazole (100%), and high sensitivity to meropenem (97.18%). Values are expressed as numbers (percentages) of isolates categorized as resistant, intermediate, or sensitive.

Drug	Resistance	Intermediate	Sensitive
Ampicillin	16 (22.53%)	9 (12.67%)	46 (64.78%)
Chloramphenicol	0	0	71 (100%)
Cotrimoxazole	0	0	71 (100%)
Ceftriaxone	0	6 (8.45%)	65 (91.55%)
Cefotaxime	0	8 (11.27%)	63 (88.73%)
Ciprofloxacin	63 (88.73%)	8 (11.27%)	0
Meropenem	0	2 (2.81%)	69 (97.18%)
Nalidix acid	71 (100%)	0	0

Azithromycin resistance was low, 2.83% (n = 10), among *S. Typhi*. However, fluoroquinolone resistance was alarmingly high: 72.23% (n = 255) of *S. Typhi* and 88.73% (n = 63) of *S. Paratyphi* were resistant to ciprofloxacin. Nalidixic acid resistance was also widespread: 94.61% (n = 334) in *S. Typhi* and 100% (n = 71) in *S. Paratyphi* (Table 9).

MDR in *S. Typhi* and *S. Paratyphi*

In our study, the prevalence of MDR in *S. Typhi* (resistant to ampicillin, chloramphenicol, and co-trimoxazole) was 3.68% (13/353; 95% CI: 1.96-6.16), whereas none of the *S. Paratyphi* isolates were MDR (Table 10).

**Table 10:** Multi-drug resistance in S. Typhi and S. Paratyphi Multi-drug resistance was observed in 3.68% (13/353) of *S. Typhi* isolates, while no *S. Paratyphi* isolate exhibited multi-drug resistance. Values are expressed as numbers and percentages of the total isolates for each serovar.

Isolate	Frequency (n = 424)	Percentage (%)
*Salmonella Typhi *(n = 353)	13	3.68%
*Salmonella Paratyphi* (n = 71)	0	0%

## Discussion

Enteric fever remains a pressing public health challenge in low- and middle-income countries, such as India, contributing substantially to morbidity and mortality despite the availability of antimicrobial therapy [6]. The increasing emergence of AMR among *Salmonella* spp. further complicates treatment approaches and underlines the necessity for continual surveillance and updated empirical guidelines [10]. Our hospital, situated in a typhoid-endemic zone and serving predominantly low-income populations, experiences a considerable burden of this disease. This study was undertaken to evaluate the clinical and microbiological profile of *Salmonella* bacteremia and assess prevailing AMR patterns, to inform empirical therapy.

Among the 16,052 blood cultures processed over 24 months, 909 (5.66%) yielded significant growth. The majority of samples were from inpatients, 14,771 (92.02%), reflecting referral trends to tertiary care centers, in contrast to studies in which outpatient samples predominated (69.44%) [11]. A slight male predominance, 8,816 (54.9%), was observed, possibly reflecting disparities in healthcare access and health-seeking behaviors between genders [12].

Typhoidal *Salmonella* spp. represented 46.64% (n = 424) of all positive isolates, indicating a prevalence of 2.64% (424/16,052) across the total sample pool. These findings are in concordance with Marchello et al. [13], though some studies cite *Klebsiella* spp. and *E. coli* as the most common pathogens [14]. Other recovered organisms included *E. coli*, 17.60%; *Staphylococcus* spp., 13.97%; *Acinetobacter* spp., 6.27%; *Klebsiella* spp., 4.51%; *Enterococcus* spp., 3.19%; *Candida* spp., 3.08%; and *Pseudomonas* spp., 2.20%, in descending order of frequency, consistent with previously reported etiological patterns in bloodstream infections. Within the typhoidal group, *S. Typhi* predominated, 83.25% (n = 353), with *S. Paratyphi* accounting for 16.74% (n = 71), including *S. Paratyphi* *B* in 1.41% (n = 6) [15]. This contrasts with findings by Malini et al., who reported a predominance of *S. Paratyphi*​​​​​​ [16].

The highest prevalence was noted among individuals aged 10-20 years (33.09%, n = 140), followed by children under 10 years (31.37%, n = 133) and young adults aged 20-30 years (26.42%, n = 112), with a mean age of 17.49 ± 10.17 years. This trend suggests increased vulnerability among children and adolescents, consistent with prior literature [17,18]. The overall male-to-female ratio was 1.51:1, supporting earlier demographic observations [18].

Fever was a universal finding (100%, n = 424). Other common symptoms included abdominal pain, diarrhea, nausea and/or vomiting, and loss of appetite, aligning with symptomatology reported in earlier studies [19]. Less frequently reported symptoms included constipation and headache. On physical examination, signs such as coated tongue, pallor, and rose spots were documented, which were similar to those reported in other studies conducted earlier [19,20]. The lower detection of rose spots may be attributable to diagnostic challenges in individuals with darker skin pigmentation [19,20]. Six patients presented with complications: five with intestinal obstruction and one with acute pyelonephritis accompanied by early shock. This rate is lower than the complication rates observed by Malini et al. [16]. Anemia, detected in 35.65% of patients, likely reflects nutritional deficiencies and socioeconomic factors prevalent in the affected population. Leukocyte count abnormalities, though not quantified in all cases, are known to occur in 15%-25% of enteric fever cases [21,22].

Changing scenarios historically, MDR *Salmonella* - resistant to ampicillin, chloramphenicol, and co-trimoxazole - led to the adoption of fluoroquinolones and, later, third-generation cephalosporins and azithromycin [20,23]. However, widespread fluoroquinolone resistance now renders them unreliable [20], and resistance to azithromycin and third-generation cephalosporins, although sporadic, is being reported [24,25]. Our study revealed MDR in 3.68% (n = 13) of *S. Typhi* isolates, with resistance to ampicillin 9.06% (n = 32), chloramphenicol 4.76% (n = 16), and co-trimoxazole 3.68% (n = 13). In *S. Paratyphi A*, ampicillin resistance was 22.53% (n = 16), while no resistance was observed for the other first-line drugs. The re-emergence of susceptibility likely reflects decreased use of these antibiotics in recent years [26]. Ciprofloxacin resistance was high: 72.23% (n = 255) in *S. Typhi *and 88.73% (n = 63) in *S. Paratyphi A*, with nearly all isolates resistant to nalidixic acid - consistent with findings of other Indian studies reporting resistance rates from 11% to 100% [27]. Fortunately, no resistance was observed for third-generation cephalosporins (ceftriaxone and cefotaxime), corroborating reports by Malini et al. and Gupta et al. [16,28]. However, a recent North Indian study indicated emerging ceftriaxone resistance of up to 3.2% in *S. Typhi* and 1.4% in *S. Paratyphi* [29]. Azithromycin resistance was observed in 2.83% (n = 10) of *S. Typhi* isolates in our study, aligning with findings by Taneja et al. (28%) [30], but contrary to other studies reporting no resistance [16,28]. No resistance was seen to meropenem, in agreement with existing literature [29].

In our study, the evolving AMR pattern among *S. enterica* serovars Typhi and Paratyphi reflects the global transition from classical MDR to fluoroquinolone resistance and emerging XDR phenotypes. Classical MDR, characterized by resistance to ampicillin, chloramphenicol, and trimethoprim-sulfamethoxazole, is primarily mediated by IncHI1 plasmids, harboring genes such as blaTEM-1, catA1, and dfrA. Although the prevalence of traditional MDR declined after reduced use of first-line agents, clonal expansion and plasmid persistence have sustained resistance in endemic regions [6].

Fluoroquinolone resistance now predominates in South Asia and is largely attributed to point mutations in the quinolone resistance-determining regions (QRDR) of gyrA and parC, along with plasmid-mediated quinolone resistance genes. The global spread of the H58 (genotype 4.3.1) lineage has significantly contributed to the stable chromosomal integration of resistance determinants and intercontinental dissemination. More concerning is the emergence of XDR strains, harboring ESBL genes such as blaCTX-M-15, conferring resistance to third-generation cephalosporins and limiting oral treatment options. Additionally, azithromycin resistance, linked to mutations in the acrB efflux pump, further threatens remaining therapeutic choices [30].

Overall, this study underscores the dynamic nature of AMR in enteric fever and the need for continuous surveillance, rational antimicrobial use, and enhanced diagnostic strategies to inform treatment and policy.

The limitations of the study were as follows: the major study population consisted of patients attending the tertiary care center, representing those with severe and typical symptoms, and underestimating self-limiting and mild cases of enteric fever in the community. Diagnosis of enteric fever was solely dependent on blood culture, which is affected by various factors such as prior antibiotic intake, timing of sample collection during the course of the disease, and the amount of sample collected for culture, ultimately affecting the sensitivity of the test and possibly resulting in a lower observed prevalence. A combination of tests, such as culture, serology, and molecular assays, would have provided more reliable results in terms of prevalence. Since the study did not include follow-up of patients after hospital discharge, we could not assess the actual outcome of the disease after treatment.

## Conclusions

This study highlights the continued burden of enteric fever in India, with *S. Typhi* as the predominant etiological agent among febrile bacteremia cases. Fever and abdominal pain were the most common presenting symptoms in typhoid patients, which remain common symptoms of other similar illnesses. This emphasizes the importance of microbiological diagnosis and evidence-based treatment for enteric fever. Antimicrobial therapy remains the mainstay of treatment. However, increasing resistance - especially to fluoroquinolones - poses a significant challenge. Given the high rates of fluoroquinolone resistance among *S. Typhi* and *S. Paratyphi A*, fluoroquinolones are no longer reliable for empirical use. Ceftriaxone, cefixime, and azithromycin remain effective treatment options in our study.

The implication of our study is that microbiological diagnosis and antimicrobial susceptibility testing are very important in the complete treatment of enteric fever patients to reduce the alarming rise in resistance and to avoid the carrier state of the disease. Given the rapidly evolving resistance landscape, control strategies must emphasize typhoid conjugate vaccination, genomic surveillance, antimicrobial stewardship, and strengthening of water and sanitation systems. 
